# Quantum dots improve peptide detection in MALDI MS in a size dependent manner

**DOI:** 10.1186/1477-3155-7-10

**Published:** 2009-12-31

**Authors:** Julian Bailes, Loïc Vidal, Dimitri A Ivanov, Mikhail Soloviev

**Affiliations:** 1 School of Biological Sciences, Royal Holloway, University of London, Egham, Surrey, TW20 0EX, UK; 2 IS2M, CNRS LRC7228, 15, rue Jean Starcky, 68057 Mulhouse, France

## Abstract

Laser Desorption Ionization Mass Spectrometry employs matrix which is co-crystallised with the analyte to achieve "soft ionization" that is the formation of ions without fragmentation. A variety of matrix-free and matrix-assisted LDI techniques and matrices have been reported to date. LDI has been achieved using ultra fine metal powders (UFMPs), desorption ionisation on silicon (DIOS), sol-gel assisted laser desorption/ionization (SGALDI), as well as with common MALDI matrices such as 2,5-dihydroxy benzoic acid (DHB), 3,5-dimethoxy-4-hydroxycinnamic acid (SA), α-cyano-4-hydroxycinnamic acid (CHCA) to name a few. A variety of matrix additives have been shown to improve matrix assisted desorption, including silicon nanowires (SiNW), carbon nanotubes (CNT), metal nanoparticles and nanodots. To our knowledge no evidence exists for the application of highly fluorescent CdSe/ZnS quantum dots to enhance MALDI desorption of biological samples. Here we report that although CdSe/ZnS quantum dots on their own can not substitute matrix in MALDI-MS, their presence has a moderately positive effect on MALDI desorption, improves the signal-to-noise ratio, peak quality and increases the number of detected peptides and the overall sequence coverage.

## Background

The term 'MALDI' (matrix assisted laser desorption ionization) was first introduced by Karas *et al. *[1] who documented the advantage of using a highly absorbing matrix that reduces the threshold irradiance required to generate ions in Laser Desorption Ionization Mass Spectrometry (LDI-MS). The presence of a matrix results in a larger degree of "soft ionization", that is the formation of ions without fragmentation. This soft laser desorption increases the ratio of molecular-to-fragment ions which is of great benefit in the detection of sample-specific ions. Matrix selection depends on the particular sample being analysed and can often be a case of trial and error to determine the one best suited, however it is typically a low molecular weight compound that is able to undergo phase transition upon excitation with laser. Because the matrix is co-crystallised with the analyte sample, this phase transition extends to the sample itself. Tanaka's "monumental blunder" when he unwittingly suspended his ultra fine metal powders (UFMPs) matrix in glycerol instead of acetone, and subsequently deviated from his standard protocol a further three times, only to stumble across a significant discovery [2] was an essential step and a breakthrough in the development of macromolecule ionization by laser irradiation. Typical chemical matrices used today are derivatives of UV absorbing organic acids such as benzoic, cinnamic or picolinic acids [3-8].

Sample excitation is usually achieved with short pulses of UV lasers in the wavelength range of 248-355 nm. Whilst 337 nm is the most commonly used wavelength for excitation with UV lasers, excitation with neodymium-doped yttrium aluminium garnet solid state Nd:YAG laser (frequency tripled to 355 nm) has been also reported for most of the common matrices. Spectrum quality generally increases with absorption and the best performance is often achieved when the excitation wavelength near matches that of the matrix absorption maxima. Dreisewerd provides a fine, extensive review on the topic for further reading [9].

Despite the application of MALDI to the analysis of a wide ranging catalogue of analytes and its relatively high tolerance of biological mixtures [10,11], the technique still suffers from a number of inherent drawbacks. The low molecular weight nature of the matrix itself means low molecular weight compounds (below ~500 m/z) are difficult to analyze because their detection is masked by the generation and detection of matrix ions. The specificity requirements of the matrix method also mean that a laborious process of trial and error may be required to ascertain the best matrix for a particular sample, as well as the optimization of analyte:matrix ratios, and co-crystallisation to avoid the formation of "hot-spots" during sample deposition if electrospray equipment is not available. MALDI is also intolerant to salt. The quest to overcome these problems has led to the development of a new line of LDI mass spectrometry techniques that do not require matrices, techniques termed matrix-free LDI-MS.

Direct LDI-MS was initially examined on a range of surfaces [12-17], but results showed that the success of this method is highly dependable on the properties of the analyte, with a high level of molecular degradation resulting from the increased laser power required. Desorption ionisation on silicon (DIOS) harnesses two useful properties of porous silicon, its ability to absorb in the UV and its physical structure, capable of trapping analytes of interest on its surface. DIOS was first reported by Wie *et al *.[18] who documents the generation of micrometer thick porous silicon layers from either n- or p-type flat crystalline silicon through an electrochemical etching process, in the presence of ethanol which helps to reduce background ion intensity.

In 2002 Lin & Chen [19] reported the development of a new technique dubbed sol-gel assisted laser desorption/ionization (SGALDI) mass spectrometry. The technique was shown to be compatible with small proteins, peptides, amino acids and small organics with detection limits stretching as far as 8.1 femtomoles. Chen & Chen [20] adopted a similar SGALDI principle to overcome sample deposition problems when using a 3,4-diaminobenzoic acid (DABA) and 3,5-DABA as matrices. Kinumi *et al *[21] investigated eleven kinds of metal particle (Al, Mn, Mo, Si, Sn, SnO_2_, TiO_2_, W, WO_3_, Zn and ZnO) in an attempt to identify promising alternatives to organic matrices. The team analysed two analytes, PEG 200 and methyl stearate. Results were encouraging, with only one of the candidates, SnO_2_, unable to ionize both PEG 200 and methyl stearate. The most impressive results were obtained with TiO_2 _powder as the matrix suspended with liquid paraffin, with which both analytes exhibited their best signal:noise ratio.

In 2003, Xu *et al *.[22] presented an interesting approach to MALDI analysis of biomolecules by using carbon nanotubes (CNTs) as a matrix. CNTs were discovered over a decade earlier [23] and have since been the subject of a wide range of experimental research. CNTs synthesized by Xu *et al *. displayed rod morphology with an overall diameter of ~20 nm each consisting of several cylindrical graphite sheets. CNTs not only absorb and transfer UV radiation to the analyte being studied, but also double as a good support for sample thus simplifying preparation procedures. A reduction in analyte fragmentation was also observed when CNTs are used as a matrix due to a lower fluence threshold. These factors, combined with the absence of any background ions from a CNT matrix, mean the method is highly useful for analysis of low molecular weight compounds, demonstrated by the successful analysis of organic compounds, β-cyclodextrin, and small peptides.

Given the surge of nanotechnology within the biological field, CNTs are unsurprisingly not the only nanostructures to be applied to MALDI analysis, silicon nanowires have also been used as a substrate for MALDI [24]. In this report the strong fluid wicking properties of SiNWs that result from their high surface area were exploited, and the chromatographic separation was combined with subsequent LDI-MS analysis of metabolites in biological samples. As with other nanostructures, a lower laser power was required in order to generate ion detection. The most reproducible of all LDI-MS approaches involving nanoparticles is that of silicon nanocavities due to the non-random nature of their synthesis [25].

Quantum dots (QDs) are highly fluorescent inorganic semiconductor nanocrystals that possess a number of unique and exciting features. The peak emission of a QD is dependent on its physical size, meaning they can be tuned to emit at any given wavelength [26], whilst it is possible to excite all QDs simultaneously with only a single short excitation wavelength. The unique and superior photophysical properties of QDs have seen them incorporated into a wide spectrum of biological applications and non-biological technologies [27-33]. Given the considerable hype surrounding the potential for QDs to revolutionize numerous areas of science, it is perhaps little surprise that they also appear to offer assistance to one of biological science's mainstream analytical techniques, MALDI TOF-MS. Matrix compounds by definition must exhibit high absorption at the excitation source wavelength, a requirement that is certainly met by QDs, all of which absorb at any wavelength below that of their emission. The application of nanodots to LDI-MS has recently been reported by way of using self-assembled germanium nanodots (GeNDs) grown on a silicon wafer and used as a matrix free method of LDI-MS (GeND-MS) for detection of peptides, proteins, synthetic oligomers, and polymer additives [34]. Useful mass spectra were obtained even for those masses under 800 m/z. Previous to this, platinum nanodots were incorporated into a novel silicon sample plate for MALDI-TOF-MS analysis of DNA [35], improving results and reproducibility. To our knowledge no evidence exists for the application of highly fluorescent CdSe/ZnS QDs to enhance MALDI desorption of biological samples.

## Methods

Alpha-cyano-4-hydroxycinnamic acid matrix solution in methanol (MALDI-QUALITY™) was from Agilent Technologies. Colloidal dispersions of CdSe/ZnS Core/Shell "EviDots" QDs in toluene were from Evident Technologies (Kit DK-C11-TOL), see Table 1 for the summary of their properties. Toluene (99.97 HPLC grade), acetone (99.5% grade) and acetic acid (Fluka (99.8% grade) were purchased from Fisher Scientific, Riedel-de Ha?n and Fluka respectively. Bovine Serum Albumin (BSA) was from PAA, Recombinant Porcine Trypsin was from Sigma. Perkin Elmer LS50B spectrofluorimeter was used to determine excitation and emission properties of the QD samples. Slit width was 5 nm and the scanning rate was 100 nm/min in all cases. Transmission electron microscope (TEM) images were obtained using a Philips CM200 microscope working at 200 kV. Specimens for the transmission electron microscopy study were prepared by the deposition of two drops of the colloidal suspensions on the surface of carbon-coated copper grids. Mass spectra were obtained using Bruker Reflex III MALDI-TOF mass spectrometer in reflectron mode. A sealed nitrogen pulsed laser (337 nm, linewidth 0.1 nm, 4 ns pulse duration, 300 microjoules rated pulse energy, average power 5 mW at 20 Hz) was used for the desorption.

**Table 1:** Properties of CdSe/ZnS Core/Shell "EviDots" quantum dots from http://www.evidenttech.com unless specified otherwise

QD	QD emission, nm^a^	QD emission, nm^b^	Emission FWHM, nm^a,c^	Estimated crystal diameter, nm^a^	Estimated molecular weight^d^, g/mol	Approx. quantum yield^a^, %
ED-C11-TOL-0520	515	517	< 35	2.1	10,000	30%-50%

ED-C11-TOL-0540	546	544	< 30	2.4	15,000	30%-50%

ED-C11-TOL-0560	561	566	< 30	2.6	23,000	30%-50%

ED-C11-TOL-0580	575	579	< 30	3.2	44,000	30%-50%

ED-C11-TOL-0600	596	598	< 30	4.0	86,000	30%-50%

ED-C11-TOL-0620	616	612	< 30	5.2, (5.05)^e^	200,000	30%-50%

^**a **^as specified by the manufacturer

^**b **^measured experimentally, using 1:100 - 1:2000 dilutions in toluene, and Perkin Elmer LS50B spectrofluorimeter (380 nm excitation wavelength).

^**c **^full width at half maximum

^**d **^for core material, excludes shell

^**e **^estimated, based on TEM data, see Figure 1.

The size of the QDs was estimated from the TEM images. The quantitative analysis of micrographs was performed in reciprocal space similarly to the classical treatment of SAXS (small-angle X-ray scattering) curves. The details of the method can be found in our previous publications [36,37]. Briefly, in the first step the micrographs were corrected for the background using home-built routines written in Igor Pro (Wavemetrics Ltd.). Further on, the two-dimensional power spectral density function (P_2_(s)) was computed from the micrographs (u(r)) up to the critical, or Nyquist, frequency as:(1)

where A denotes the image area, W(r) window function [38] and s the 2D reciprocal space vector. The P_2_(s) function was then transformed into the one-dimensional PSD (P_1_(s)), where s stands for the norm of s, according to:(2)

For quantitative analysis of circular-shaped objects observed in our experiments, the radial one-dimensional correlation function was calculated. This function is defined as:(3)

and can be readily calculated in its isotropic form (i.e. as a function of the norm of R) from the corresponding one-dimensional spectral density:(4)

where J_0 _is the zero-order Bessel function.

Matrix was prepared by mixing 180 ul of the alpha-cyano-4-hydroxycinnamic acid matrix stock solution in methanol, 90 ul acetone and 3 ul 1% acetic acid in a glass vessel. Matrix solution without QDs was designated "QD0" and used as it is. Three more matrix mixtures were prepared by adding × 5 ul of each of the two QD colloidal dispersions to 50 ul of the prepared matrix in 200 ul polypropylene tubes and used immediately. Table 2 summarises the matrix solutions made. Proteolytic digestion was with Trypsin, BSA was mixed with enzyme at 10:1 (by weight) in 20 mM potassium phosphate buffer (pH 9.0) and incubated for 10 hours at 37°C. The digest was used for MALDI-TOF-MS without further purification. Digested BSA samples were mixed with the matrices in 200 ul polypropylene tubes by adding 1 ul of protein to 10 ul of matrices (QD0, QD1, QD2 and QD3), re-suspending with a hand pipette and immediately spotting onto stainless steel MALDI plate. Spectra were acquired with 30 laser shots (at 10 Hz) using a range of laser settings (typically between 20 and 60% power setting). All spectra were calibrated using BSA peptides as internal standards by running "Calibrant assignment" quadratic calibration in the "Auto assign" mode. Numbers of automatically assigned calibrants were recorded for each spectrum. Mascot search engine http://www.matrixscience.com and SwissProt database were used for peptide mass fingerprinting (taxonomy - mammals, peptide tolerance +/- 200 ppm and 1 missed cleavage was allowed). All the parameters (spectra acquisition, analysis, peptide mass fingerprinting) were identical for all samples in all experiments.

**Table 2:** Quantum dot mixtures used to assist MALDI-TOF analysis

QD mixture	CdSe/ZnS Core/Shell "EviDots" quantum dots used (mixed 50:50% v/v)^b^	**QD emissionof the mixtures, nm^c^**
QD0	none added	n/a

QD1	ED-C11-TOL-0520 (517 nm) + ED-C11-TOL-0540 (544 nm)	519 nm (Ex = 337 nm)^d^ 520/539 nm (double peak, Ex = 380 nm)

QD2	ED-C11-TOL-0560 (566 nm) + ED-C11-TOL-0580 (579 nm)	575 nm (Ex = 337 nm)^d^ 575 nm (Ex = 380 nm)

QD3	ED-C11-TOL-0600 (598 nm) + ED-C11-TOL-0620 (615 nm)	614 nm (Ex = 337 nm)^d^ 614 nm (Ex = 380 nm)

^**a **^see Methods for further details

^**b **^emission measured using excitation at 380 nm

^**c **^excitation wavelength as specified

^**d **^excitation at 337 nm corresponds to the wavelength of the nitrogen laser used for MALDI experiments

## Results

### 1. Characterisation of quantum dot preparations

**Figure 1:**
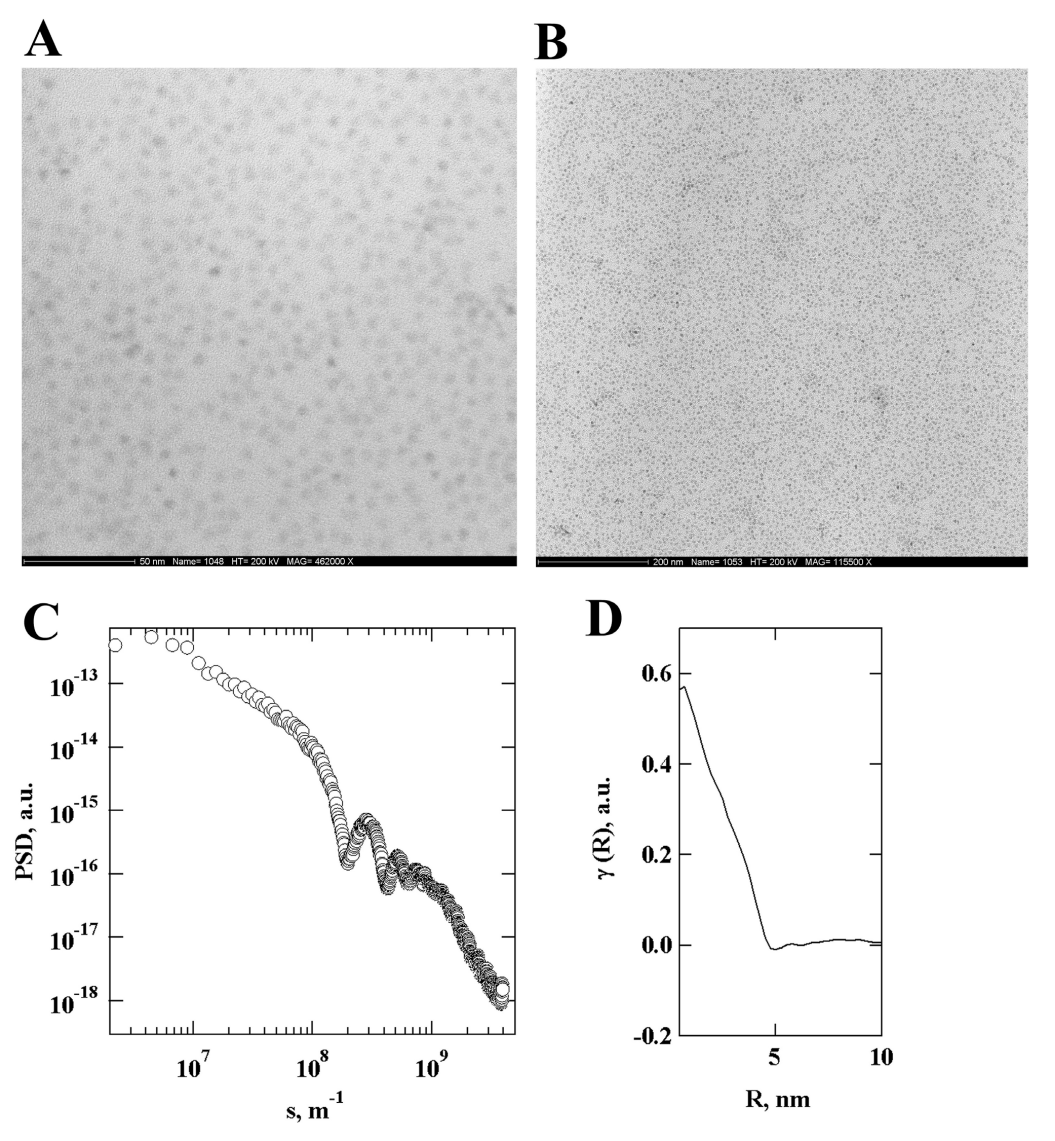
**TEM analysis of CdSe/ZnS Core/Shell "EviDots" (Evident Technologies) deposited on the surface of carbon-coated copper grids**. Panels (A) and (B) exemplify the results obtained for ED-C11-TOL-0620 "EviDots". TEM images were used to determine the size of the quantum dots. Panel (C) illustrates power spectral density function (PSD) corresponding to image on panel (B). The corresponding radial correlation function (D) allows estimating the average diameter of the particle (5.05 nm).

CdSe/ZnS Core/Shell "EviDots" from Evident Technologies were first characterised by TEM imaging. Figure 1 (Panels A and B) illustrates an example of the images obtained (shown for ED-C11-TOL-0620 QDs). TEM images obtained for all other "EviDots" preparations are provided as Supporting Information (see Additional files 1, 2, 3, 4, 5 and 6). TEM images allowed the size of the QD to be determined. Figure 1 (Panels C) shows a typical power spectral density function computed from the TEM micrograph shown on Panel B (Figure 1). The PSD function reveals several ripples indicating that the studied nanoparticles are highly monodisperse. The lateral size of the nanoparticles has been calculated from the corresponding one-dimensional radial correlation function (Figure 1, Panels D) as the extrapolation of the linear slope in the self-correlation triangle to the intersection with the baseline. The estimated size of the ED-C11-TOL-0620 "EviDots" was 5.05 nm, which agrees well with the values supplied by the manufacturer (see Table 1), other "EviDots" preparations were not analysed. Fluorescent excitation and emission spectra were taken for each individual QD sample. Table 1 summarises the emission maxima obtained for each QD sample.

### 2. CdSe/ZnS quantum dots on their own can not substitute matrix in MALDI

**Figure 2:**
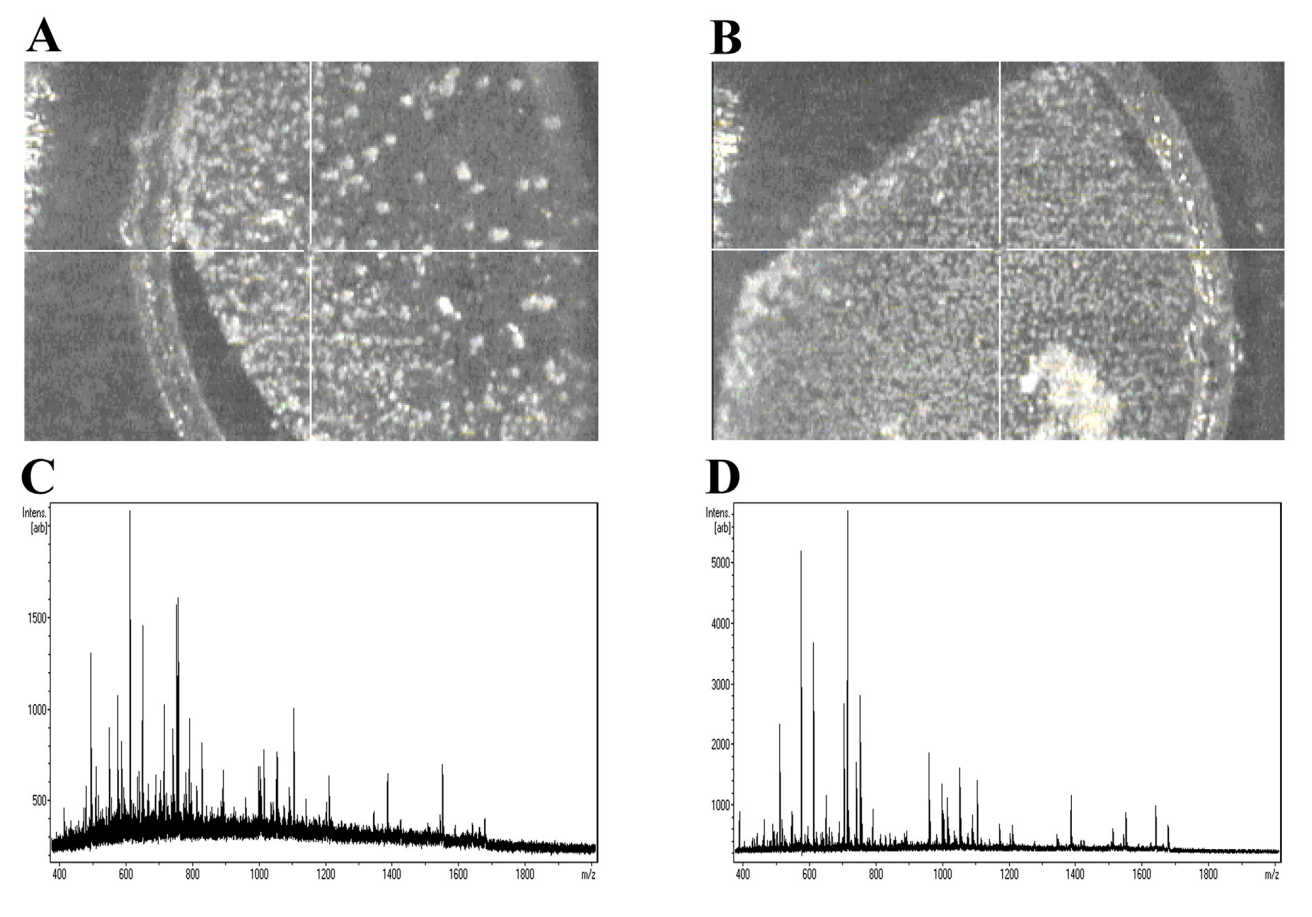
**Crystallised MALDI matrix (Alpha-cyano-4-hydroxycinnamic acid)**. (A) Tryptic digest of BSA (no purification, no quantum dots). (B) Tryptic digest of BSA (same as in Panel A), containing 50:50% mixes (v/v) of ED-C11-TOL-0560 (566 nm) and ED-C11-TOL-0580 (579 nm) "EviDots". (C) Typical MALDI spectra obtained from tryptic digests of BSA spotted with matrix only or matrix mixed with "EviDots" (D). The amount of protein and the matrix spotted are the same in both cases. Spectra were acquired with 30 laser shots (at 10 Hz) using 50% laser power setting in both cases. In Panel (D) the matrix solution contained a mixture of ED-C11-TOL-0520 (517 nm) and ED-C11-TOL-0540 (544 nm) "EviDots".

The use of self-assembled germanium nanodots (GeNDs) and Pt nanodots have been reported previously [34,35] for the detection of peptides, proteins, synthetic oligomers and DNA with MALDI-TOF-MS, but no reports on CdSe/ZnS are yet available. We therefore started by applying CdSe/ZnS Core/Shell "EviDots" (Evident Technologies) QDs instead of a standard alpha-cyano-4-hydroxycinnamic acid matrix solution. Six different QD samples were tested individually and various laser power settings were used (from 0 to 100%) but no peaks were detected when Trypsin digest of BSA was spotted and analysed (data not shown). We do not exclude a possibility that the use of other solvents or glycerol might recover the ability of CdSe/ZnS QDs to act as matrix, but since the use of glycerol, its advantages and disadvantages have been reported previously [39-43] we have not pursued this line of investigation. We have decided, instead, to use QDs together with the standard alpha-cyano-4-hydroxycinnamic acid matrix. We prepared three different QD mixtures and analysed them separately along with the matrix-only (sample QD0). All matrix/QD samples contained a mixture (50:50% v/v) of two QD preparations, see Table 2 for the summary and nomenclature. QDs were added to the matrix stock solution prior to the protein digest, as described in the Methods. Following the spotting, we noticed that the visual appearance of the spotted samples changed, see Figure 2. All of the samples containing QDs appeared to have somewhat smaller and more uniform matrix crystals and many (but not all) had small patches of apparently more densely clustered crystals. The spots with patches yielded similar MALDI spectra from patches to the ones obtained when desorption was from outside those areas (data not shown). However, for the reasons of consistency these dense patches were, where possible, avoided when spectra were acquired for comparing matrix only vs. matrix/QD preparations. Surprisingly, MALDI spectra generated from the "matrix/QD" spots appeared to contain stronger peaks, required lower laser power to obtain them and appeared to have lower "noise", as shown on Figure 2. We therefore endeavoured to quantify these and any other differences.

### 3. The presence of CdSe/ZnS quantum dots in alpha-cyano-4-hydroxycinnamic acid matrix facilitates MALDI desorption, improves the signal-to-noise ratio and peak quality

**Figure 3:**
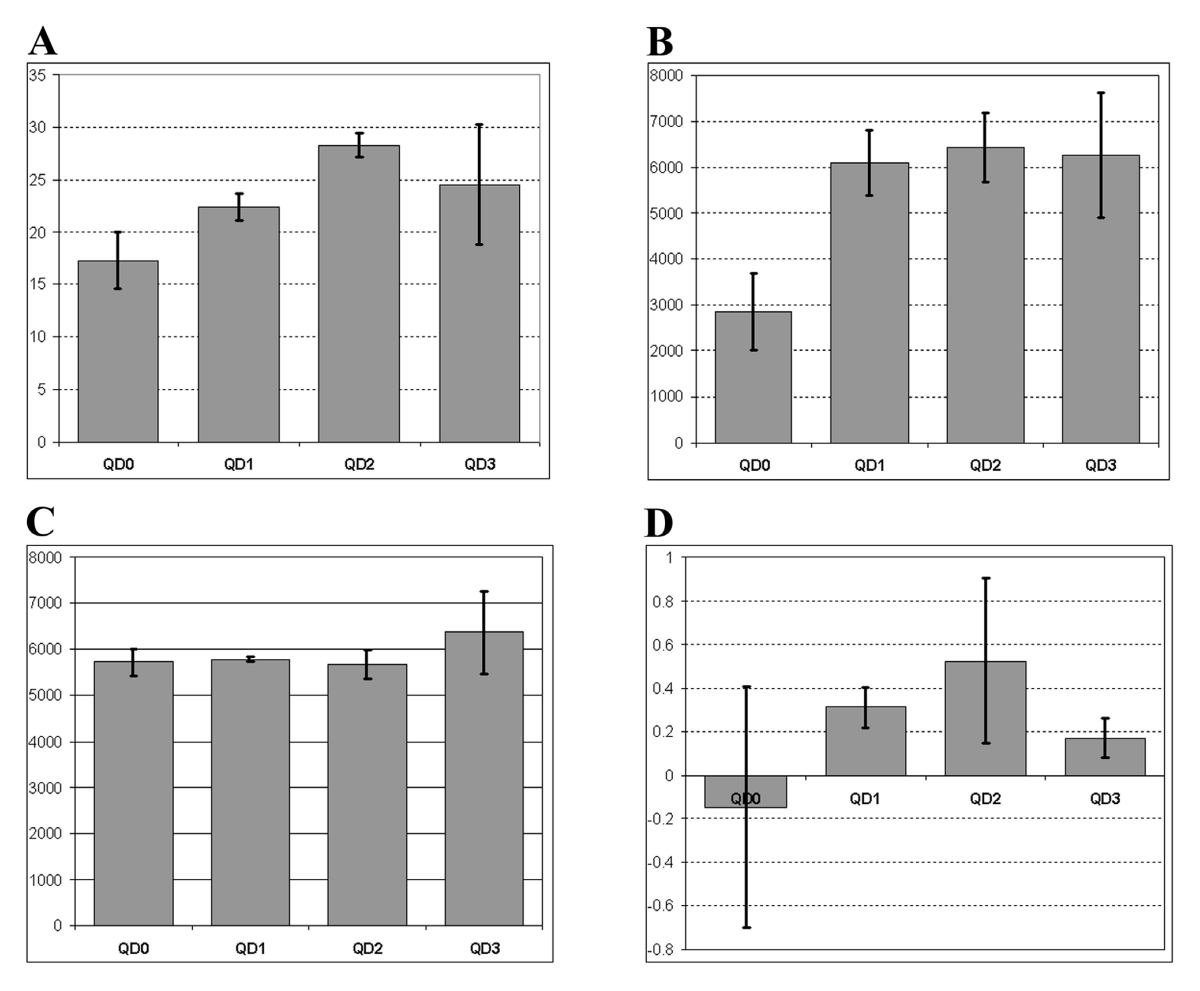
**The analysis of MALDI spectra: peaks' statistics**. Peak lists were generated automatically for each spectra taken using FlexAnalysis v.2.4 supplied by Bruker Daltonics. (A) Signal-to-noise values (S/N) for each individual peak were extracted from all of the mass spectra taken for each individual preparation in each experiment. (B) Peak quality factor (QF) values, as reported by the FlexAnalysis software. Analysed similarly to S/N values. (C) Peak resolution values (Res), as reported by the FlexAnalysis software. Panel (D) shows the skew of the Res distribution for each matrix/QD preparation. In all panels, all values were averaged over all the peaks from all the spectra (for each particular matrix/QD preparation) from each individual experiment, and are shown as means +/- STDEV, from 3 experiments for each matrix/QD preparation.

Signal-to-noise values (S/N) for each individual peak were extracted from the list of automatically generated peaks from all of the mass spectra taken for each individual preparation in each experiment (multiple spectra for each of the QD mix preparation were taken, including the matrix only and all three different matrix/QD preparations). Whereas S/N values within each individual spectrum varied significantly (ranging between ~5 and ~190), the S/N values averaged over all the peaks from all the spectra (for a particular QD preparation) from an individual experiment were less variable if compared between the experiments (for the same QD preparations). The graph on the figure Figure 3, (panel A) shows mean S/N values for each individual preparation further averaged over three experiments and expressed as (+/- STDEV, n = 3 experiments). All of the QD-containing preparations showed higher S/N values with the QD2 samples showing significantly higher S/N values than the ones obtained without any QDs added (QD0). We have also analysed peak quality factor (QF) values (these are different form the "resolution" values), which were similarly extracted from the list of automatically generated peaks by the "FlexAnalysis" software from all of the mass spectra and analysed them similarly to the S/N values. All of the samples containing QDs showed significantly higher QF values than the ones obtained from the matrix alone spectra, see Figure 3, (panel B). Interestingly the detected resolution values (Res) of the peaks were not significantly different between the samples tested, when analysed in a similar manner to the analysis of S/N and QF values described above, see Figure 3, (panel C). However we noticed that the skew of the Res distribution changed noticeably, see Figure 3, (panel D). Our data indicate that the peaks recorded with the QDs added contained larger proportion of higher resolution peaks than the samples without QDs. Because the mean values remained the same, it would also mean that QD samples must have had a proportion of peaks with resolution lower than achieved without QDs added. Since the recorded Res values and the skew changed insignificantly between the samples, this phenomenon may require further investigation to conclude whether QDs have any real effect on resolution or not.

### 4. The presence of CdSe/ZnS quantum dots in alpha-cyano-4-hydroxycinnamic acid matrix enhances peptide desorption and increase the number of detected peptides and the overall sequence coverage

**Figure 4:**
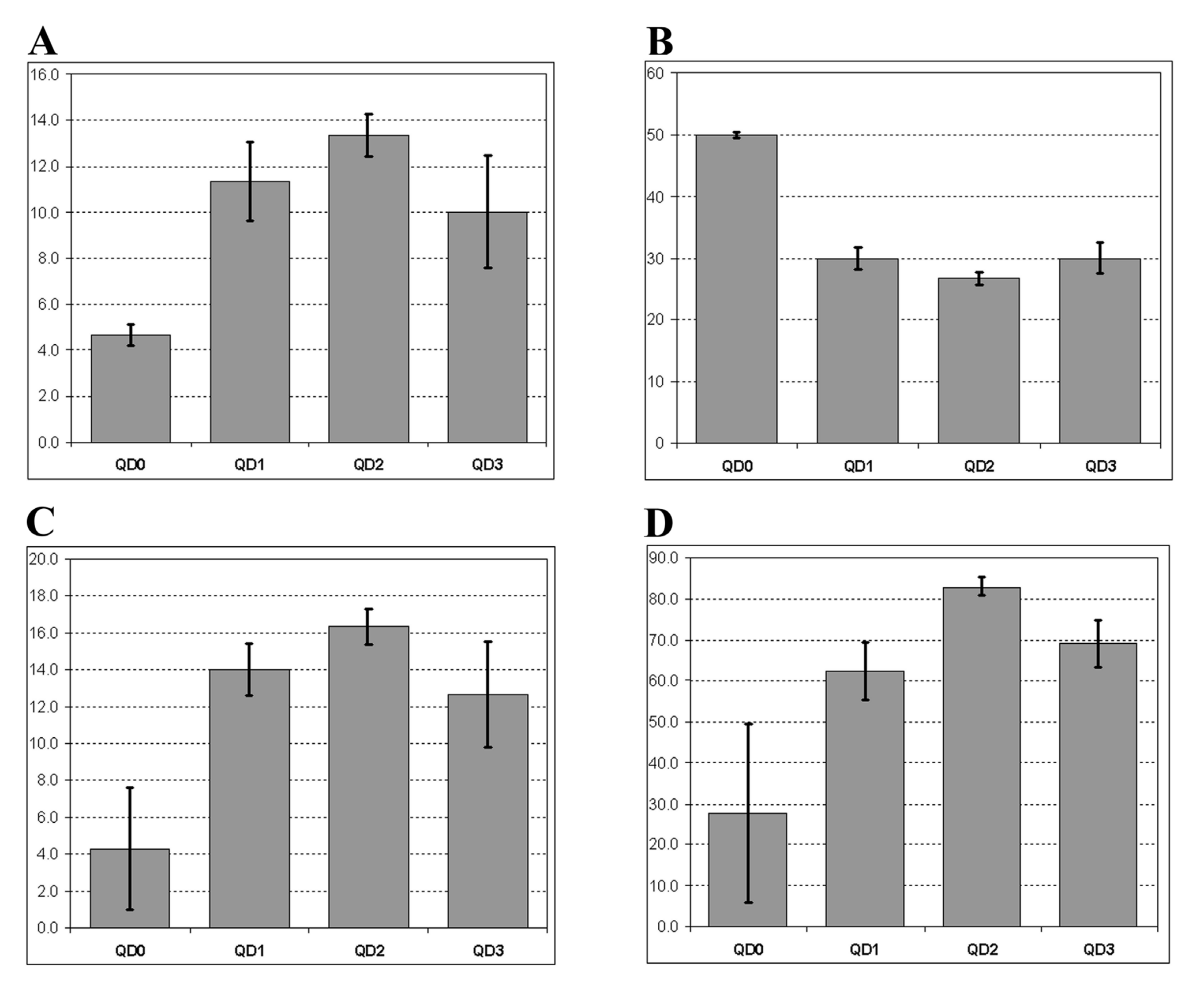
**The analysis of MALDI spectra: mass spectra calibration and mass matching**. (A) Automatic peak assignment for all spectra using the list of BSA/trypsin masses and the "Auto Assign" function of the "FlexAnalysis" software. Vertical axis indicates the number of peaks automatically assigned in each case (mean +/- STDEV, from 3 experiments). (B) Minimum laser power setting for each matrix/QD preparation (from each individual experiment), at which automatic calibration was possible (mean +/- STDEV, from 3 experiments). (C) the highest number of BSA masses identified by MASCOT http://www.matrixscience.com and the "mascot scores" (D) averaged over all spectra for each individual matrix/QD sample and shown as mean +/- STDEV, from 3 experiments.

We used the same sample of tryptically digested bovine serum albumin (BSA) in all experiments to avoid any sample variability between spots and samples. Therefore, should different number of peptides be detected, such an effect would be due to differences in the desorption rather than peptide sample content variability. In order to objectively compare the number of *detectable *peaks we relied on the automatic peak assignment ("Auto Assign" function) by the mass spectrum calibration feature of the "FlexAnalysis" software fed with a complete list of BSA tryptic peptides (fully digested, no missed cleavages). The software will automatically search the mass list of the selected mass spectrum for current masses and identify all suitable peptide peaks (quality, resolution, intensity, and m/z values that differ from the reference mass by not more than a user-defined value) and hence the number of peptides used for the calibration can be used as an indirect indication of how many peaks of acceptable quality were present within the tolerance range specified. Because all samples in all experiments were analysed using a variety of settings, including different setting of the laser power and different positions within each spot we used for our analysis the *maximum *values of the calibration peptide peaks from all the mass spectra obtained for each matrix/QD mix. These were then averaged over all the experiments and plotted as means (+/- STDEV, n = 3 experiments each), see Figure 4 (panel A). The increase in the number of identified peaks (suitable for calibration) is significant in all QD-containing samples. Lower number of peaks identified in matrix only samples does not mean that no other BSA masses were detected (more were present, as identified by MASCOT, see below), but shows that fewer high quality peaks were available. Furthermore, since a range of laser power setting was used in each experiment for each matrix/QD sample, we were able to compare the minimum laser power settings at which automatic spectrum calibration was possible (i.e. three or more suitable peaks were present). The results are summarised in panel B, Figure 4. The automatic calibration was achieved at significantly lower laser power settings in all QD-containing samples. Automatic calibration was almost never achieved with matrix only samples at laser power less than 50%. The absolute laser power may be difficult to assess and it may differ significantly between the instruments and individual lasers, but the relative values obtained with the same instrument reveal quantitative differences. It is important to note here that manual calibration was possible even at lower power settings for all samples, including matrix only, but the manual approach would be subjective hence was not used.

Following the calibration (all spectra were calibrated using internal standards) and the de-isotoping, the peaks were automatically extracted using "FlexAnalysis" software and the mass-to-charge ratios (m/z) were submitted to the MASCOT search. Significantly more BSA masses were identified in all QD-containing samples. Results are summarised in Figure 4 (Panel C), which shows the maximum numbers of BSA masses identified by MASCOT from all spectra for each matrix/QD samples (the sequence coverage similarly increased, data not shown). Similarly to the S/N, QF and Res analyses described earlier, the values here are means from different experiments and the STDEV values shown indicate variability between the experiments and not between the values from different spectra for the same matrix/QD sample from the same experiment (the latter are highly variable since different laser power settings were used). The larger number of the identified masses resulted in noticeably higher MASCOT scores obtained for the samples containing QDs, see Figure 4 (Panel D). The use of QD2 matrix mixture yielded the highest MASCOT scores. In the absence of QDs, variability in the scores is significantly higher than in all other cases investigated. The "Expect" values reported by MASCOT (the probability that the match was a random event of no significance) were also different. The overall best (minimum) "Expect" value over all experiments, samples and spectra for the different QD samples was recorded for QD2 (p = 0.00014, i.e. over ×300 fold lower than the default Mascot value of 5%).

## Discussion

A number of additives are documented in the literature, the presence of which assists the matrix in achieving successful desorption/ionization of the analyte. Those mentioned so far include glycerol [39], used to ensure a uniform analyte matrix mixture is achieved and also aids release of the sample from its crystalline state, and NIPPOLAN-DC-205 [44] used for immobilizing carbon nanotubes to the target plate. Glycerol has also performed well when used for sample desorption with infra-red solid state lasers (Er-YAG and Er-YSGG) [43]. Paraffin was utilized in sample preparation by Kinumi *et al *[21] for the analysis of small molecules. The inclusion of paraffin was shown to greatly reduce low molecular mass noise from matrix ions, which in turn permitted the improved study of small molecules with inorganic metal particle matrix. Cu(II) ions have also been successfully used as electron scavengers in MALDI to prevent the reduction of analytes that can impair analysis [45]. Other additives include those such as ammonium citrate to improve spectral quality for protein digest analysis with DIOS [46]. Platinum nanoparticles have been reported to improve MALDI results and [35] and self-assembled germanium nanodots (GeNDs) were shown to desorb peptides, proteins, synthetic oligomers, and polymer additives without any matrix added [34].

In our hands the colloidal dispersions of CdSe/ZnS Core/Shell "EviDots" QDs did not work without the matrix, but improved the performance of MALDI-TOF-MS when co-applied together with the alpha-cyano-4-hydroxycinnamic acid matrix. We can not be certain as to the exact mechanism of this effect and a number of explanations remain possible. On drying, the dots may have formed a sol-gel and increased the surface area at which the desorption occurred, thus increasing the efficiency of MALDI. Similar effects have been reported previously, e.g. SGALDI mass spectrometry using polymeric sol-gels with 2,5-Dihydroxybenzoic acid (DHB), 3,4-diaminobenzoic acid (DABA) and 3,5-DABA as matrices [19,20,47], or with titanium based sol-gels [48,49]. Alternatively, the addition of QDs could have helped matrix crystallisation, by serving as nucleation centres for the growing matrix crystals. Another plausible explanation, from our point of view, is that the addition of QDs resulted in an adjustable red-shift of the 337 nm wavelength of the nitrogen laser and in the case of the best performing QD2 mix (with the Emission maxima at 561 nm and 579 nm (575 nm, when mixed at 50:50% v/v) could have improved the photon absorption by the alpha-cyano-4-hydroxycinnamic acid matrix. It has been reported that alpha-cyano-4-hydroxycinnamic acid matrix is suitable for use with both nitrogen laser (337 nm) [39] and the neodymium-doped yttrium aluminium garnet solid state Nd:YAG laser (355 nm) [1].

The maximum absorption for alpha-cyano-4-hydroxycinnamic acid in methanol is 340 nm (50 nm full width at half maximum) and the absorption peak does not extend into the area where main emission peaks of the "EviDots" are. At the same time no noticeable Emission was detected for any of the "EviDots" preparations below ~450 nm. However, when crystallised both the alpha-cyano-4-hydroxycinnamic acid absorption properties as well as QDs fluorescent properties could have changed. In our experiments we used nitrogen laser (337 nm) and the addition of QDs must have red-shifted the laser energy and the broader illumination spectrum must have better matched the absorption spectrum of the crystallised matrix. Clear distinction between the three QD preparations with the QD2 mixture (575 nm emission wavelengths) having the strongest effect supports the size and/or wavelength specific QDs effect on MALDI desorption. It is not impossible that all the above factors acted in concert and we do not exclude the possibility that another additional mechanism exist by which ED-C11-TOL-0560 and ED-C11-TOL-0580 "EviDots" specifically enhance MADLI desorption.

## Conclusions

Here we report that CdSe/ZnS QDs have a moderately positive effect on MALDI desorption of crude tryptic digests by improving the signal-to-noise ratio, peak quality and increasing the number of detected peptides and the overall sequence coverage. CdSe/ZnS QDs on their own can not substitute matrix in MALDI-MS as no spectra were obtained in the absence of alpha-cyano-4-hydroxycinnamic acid matrix. We conclude therefore that the use of fluorescent quantum dots in addition to standard MALDI matrix may further improve the technique of MALDI-TOF-MS and extend the range of usable matrices. However, further work might be required to optimise the solvents, QD composition, QD-to-matrix ratios and QDs emission wavelengths

## Competing interests

The authors declare that they have no competing interests.

## Authors' contributions

All authors contributed equally, read and approved the final manuscript.

## Supplementary Material

Additional file 1 **ED-C11-TOL-0520 "EviDots"**. TEM analysis of CdSe/ZnS Core/Shell ED-C11-TOL-0520 "EviDots" (Evident Technologies) deposited on the surface of carbon-coated copper grids. Click here for file

Additional file 2 **ED-C11-TOL-0540 "EviDots"**. TEM analysis of CdSe/ZnS Core/Shell ED-C11-TOL-0540 "EviDots" (Evident Technologies) deposited on the surface of carbon-coated copper grids. Click here for file

Additional file 3 **ED-C11-TOL-0560 "EviDots"**. TEM analysis of CdSe/ZnS Core/Shell ED-C11-TOL-0560 "EviDots" (Evident Technologies) deposited on the surface of carbon-coated copper grids. Click here for file

Additional file 4 **ED-C11-TOL-0580 "EviDots"**. TEM analysis of CdSe/ZnS Core/Shell ED-C11-TOL-0580 "EviDots" (Evident Technologies) deposited on the surface of carbon-coated copper grids. Click here for file

Additional file 5 **ED-C11-TOL-0600 "EviDots"**. TEM analysis of CdSe/ZnS Core/Shell ED-C11-TOL-0600 "EviDots" (Evident Technologies) deposited on the surface of carbon-coated copper grids. Click here for file

Additional file 6 **ED-C11-TOL-0620 "EviDots"**. TEM analysis of CdSe/ZnS Core/Shell ED-C11-TOL-0620 "EviDots" (Evident Technologies) deposited on the surface of carbon-coated copper grids. Click here for file
