# Supplementary figures and images for: Quantum dots improve peptide detection in MALDI MS in a size dependent manner
Source: J Nanobiotechnology. 2009 Dec 31;7:10. doi: 10.1186/1477-3155-7-10 (PMC2806340; doi:10.1186/1477-3155-7-10)

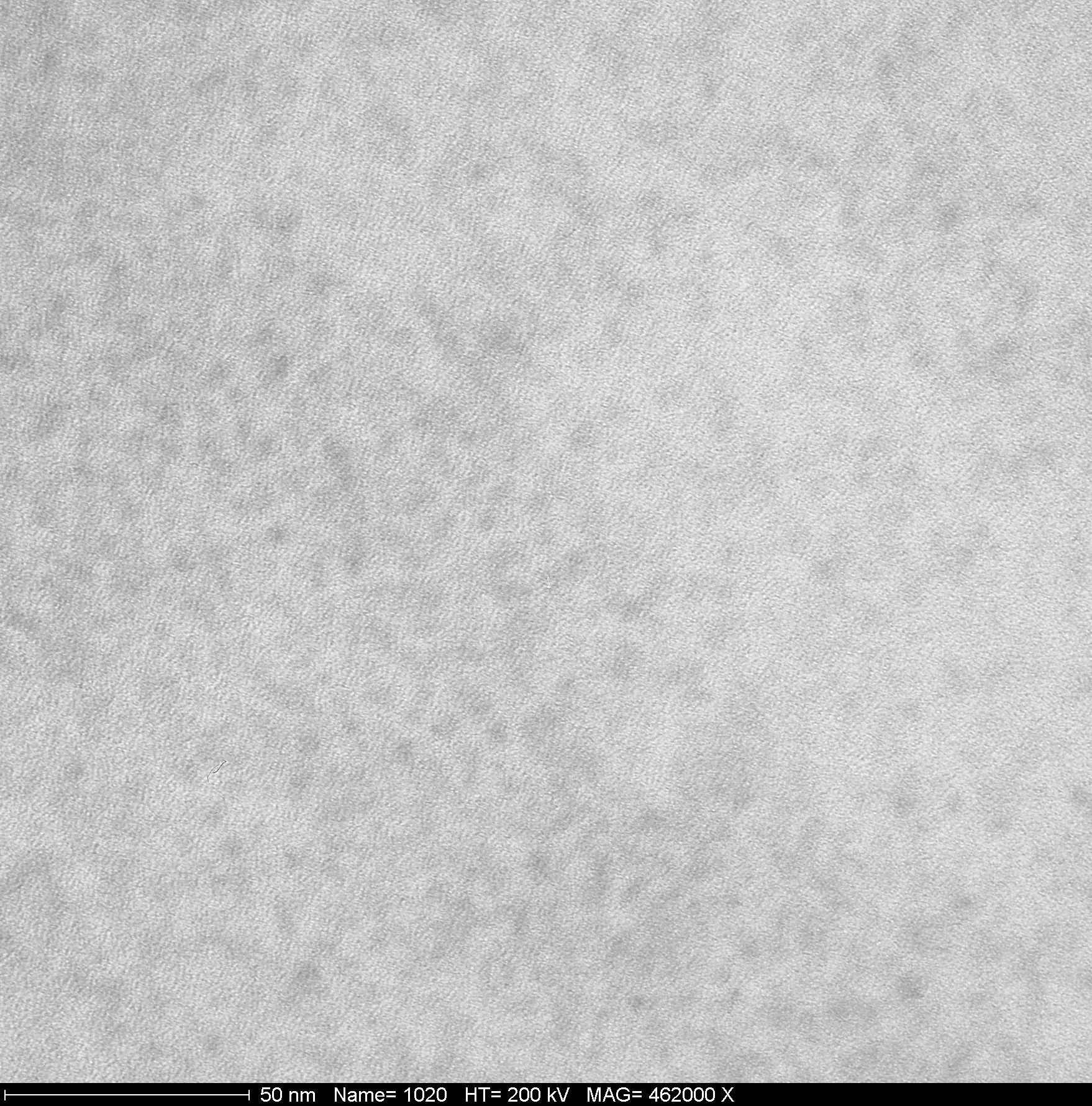

Supplement: Additional file 1 — ED-C11-TOL-0520 "EviDots". TEM analysis of CdSe/ZnS Core/Shell ED-C11-TOL-0520 "EviDots" (Evident Technologies) deposited on the surface of carbon-coated copper grids. [file 1477-3155-7-10-S1.JPEG]

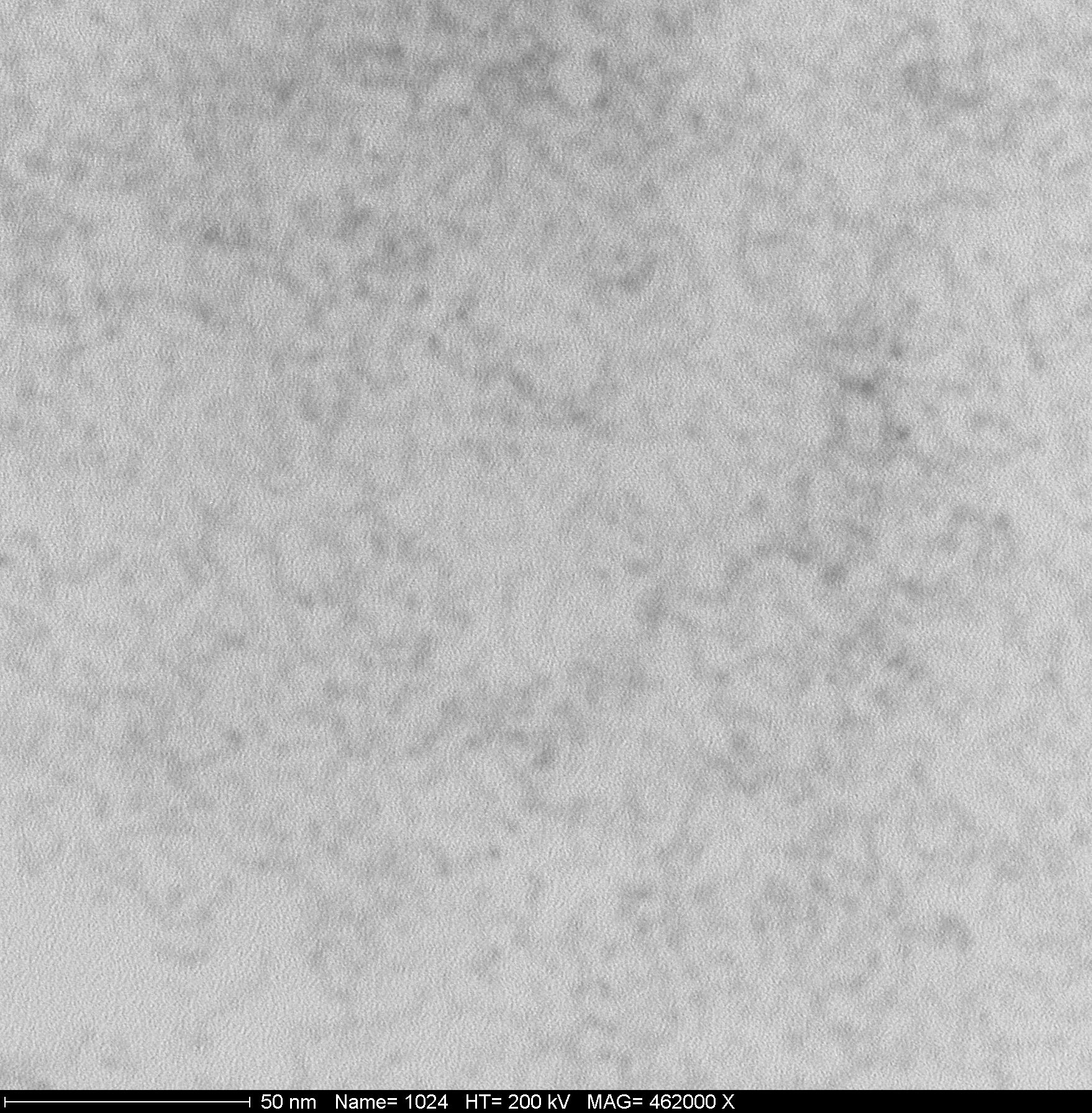

Supplement: Additional file 2 — ED-C11-TOL-0540 "EviDots". TEM analysis of CdSe/ZnS Core/Shell ED-C11-TOL-0540 "EviDots" (Evident Technologies) deposited on the surface of carbon-coated copper grids. [file 1477-3155-7-10-S2.JPEG]

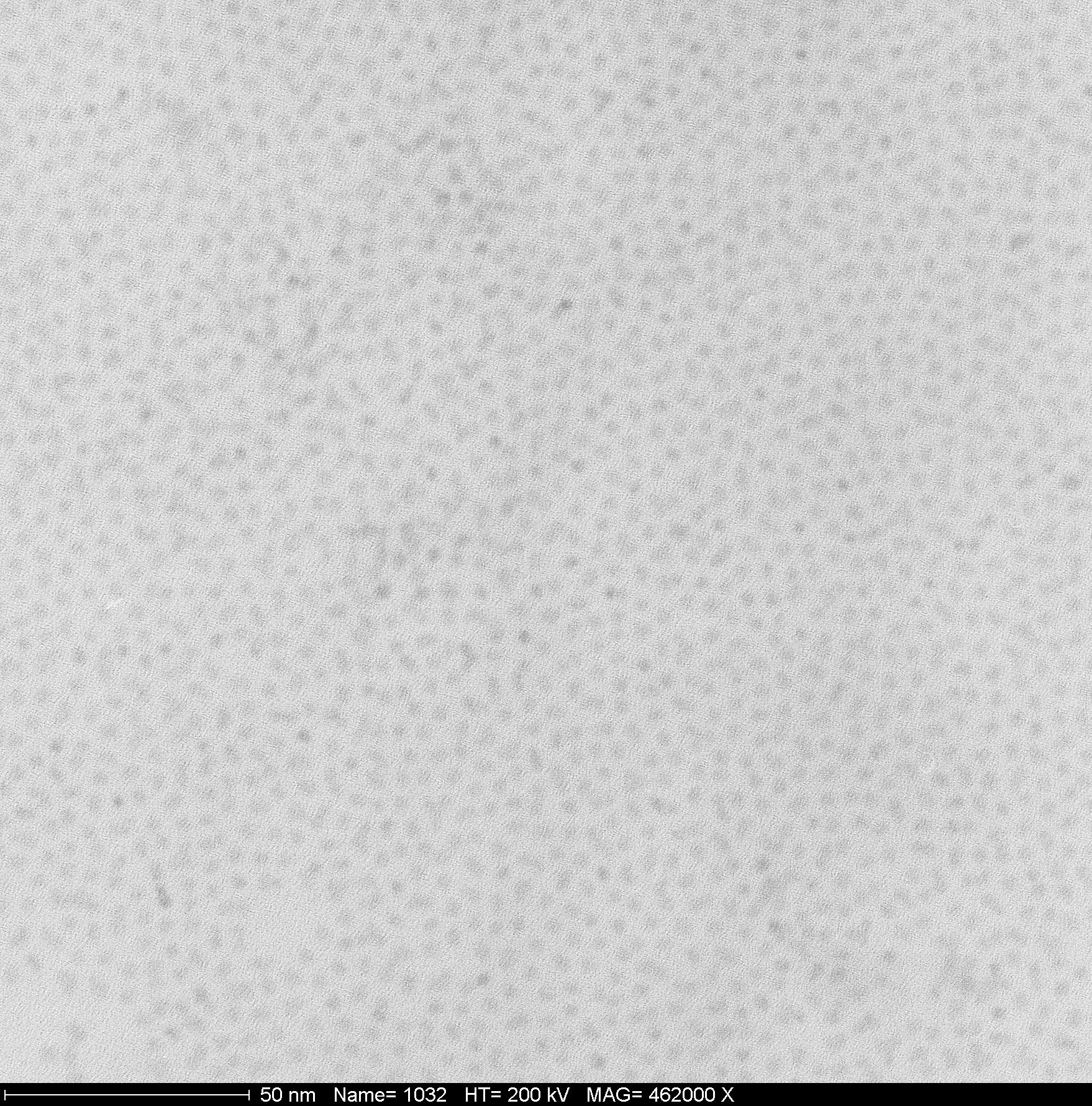

Supplement: Additional file 3 — ED-C11-TOL-0560 "EviDots". TEM analysis of CdSe/ZnS Core/Shell ED-C11-TOL-0560 "EviDots" (Evident Technologies) deposited on the surface of carbon-coated copper grids. [file 1477-3155-7-10-S3.JPEG]

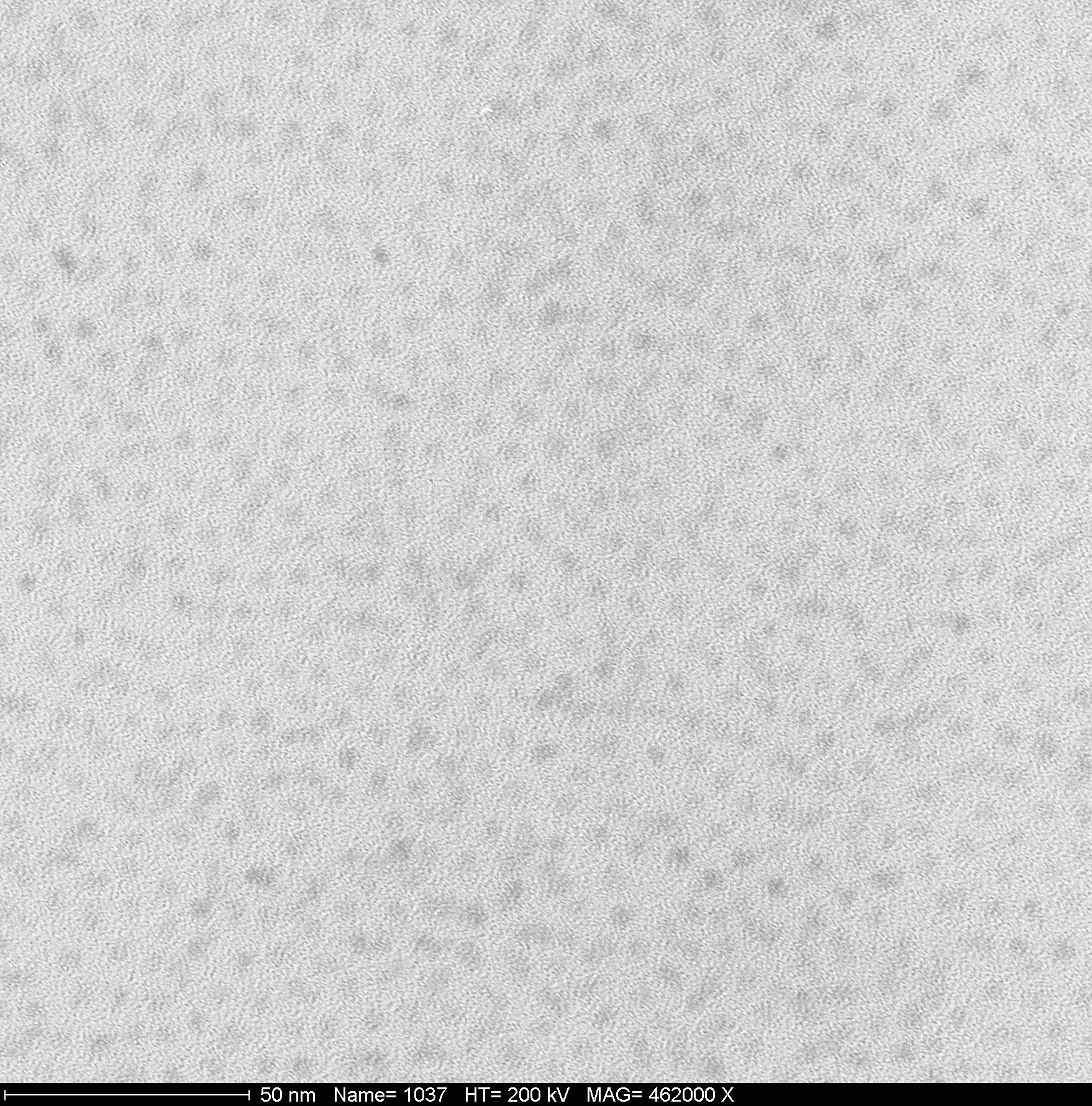

Supplement: Additional file 4 — ED-C11-TOL-0580 "EviDots". TEM analysis of CdSe/ZnS Core/Shell ED-C11-TOL-0580 "EviDots" (Evident Technologies) deposited on the surface of carbon-coated copper grids. [file 1477-3155-7-10-S4.JPEG]

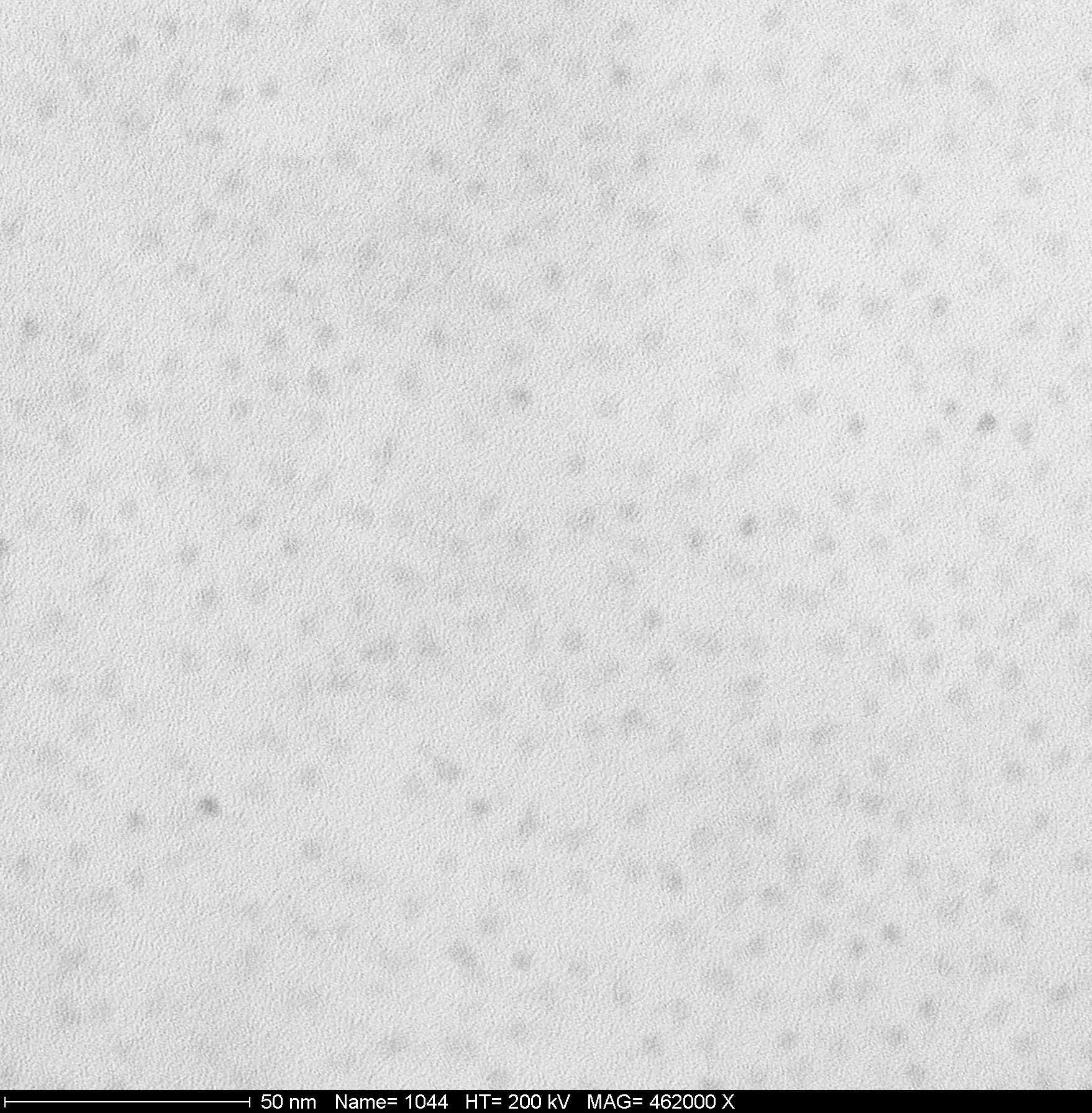

Supplement: Additional file 5 — ED-C11-TOL-0600 "EviDots". TEM analysis of CdSe/ZnS Core/Shell ED-C11-TOL-0600 "EviDots" (Evident Technologies) deposited on the surface of carbon-coated copper grids. [file 1477-3155-7-10-S5.JPEG]

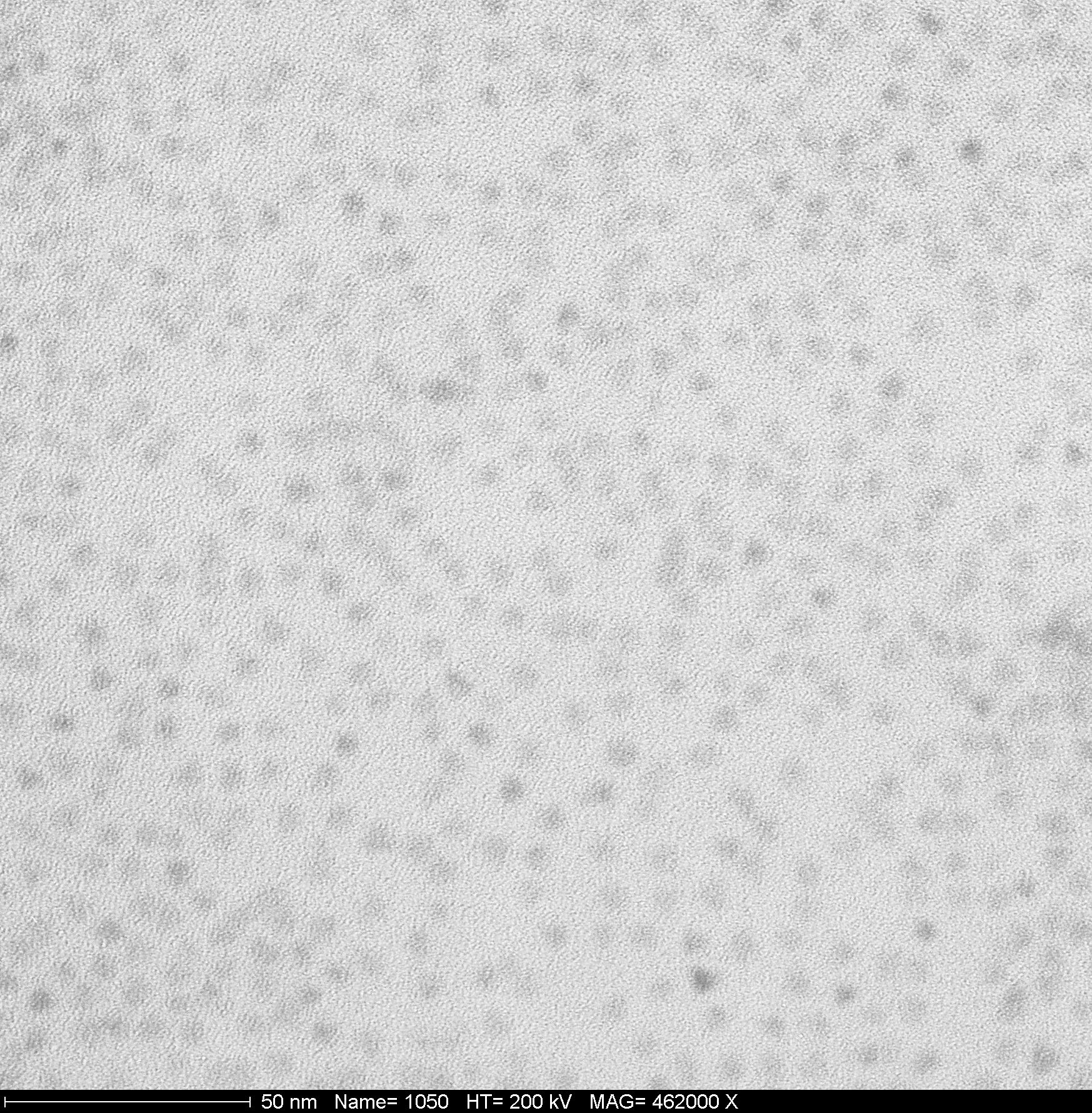

Supplement: Additional file 6 — ED-C11-TOL-0620 "EviDots". TEM analysis of CdSe/ZnS Core/Shell ED-C11-TOL-0620 "EviDots" (Evident Technologies) deposited on the surface of carbon-coated copper grids. [file 1477-3155-7-10-S6.JPEG]
